# Morbid Sequences Suggest Molecular Mimicry between Microbial Peptides and Self-Antigens: A Possibility of Inciting Autoimmunity

**DOI:** 10.3389/fmicb.2017.01938

**Published:** 2017-10-09

**Authors:** Susanta Pahari, Deepyan Chatterjee, Shikha Negi, Jagdeep Kaur, Balvinder Singh, Javed N. Agrewala

**Affiliations:** ^1^Immunology Laboratory, CSIR-Institute of Microbial Technology, Chandigarh, India; ^2^Department of Biotechnology, Panjab University, Chandigarh, India

**Keywords:** molecular mimicry, microbes, autoimmunity, autoantigens, immunoinformatics, HLA binders, sequence and structural mimicry, cytokines

## Abstract

Understanding etiology of autoimmune diseases has been a great challenge for designing drugs and vaccines. The pathophysiology of many autoimmune diseases may be attributed to molecular mimicry provoked by microbes. Molecular mimicry hypothesizes that a sequence homology between foreign and self-peptides leads to cross-activation of autoreactive T cells. Different microbial proteins are implicated in various autoimmune diseases, including multiple sclerosis, human type 1 diabetes, primary biliary cirrhosis and rheumatoid arthritis. It may be imperative to identify the microbial epitopes that initiate the activation of autoreactive T cells. Consequently, in the present study, we employed immunoinformatics tools to delineate homologous antigenic regions between microbes and human proteins at not only the sequence level but at the structural level too. Interestingly, many cross-reactive MHC class II binding epitopes were detected from an array of microbes. Further, these peptides possess a potential to skew immune response toward Th1-like patterns. The present study divulges many microbial target proteins, their putative MHC-binding epitopes, and predicted structures to establish the fact that both sequence and structure are two important aspects for understanding the relationship between molecular mimicry and autoimmune diseases. Such findings may enable us in designing potential immunotherapies to tolerize autoreactive T cells.

## Introduction

Autoimmune diseases are highly debilitating ailments that have shown sharp increase worldwide. Over 100 million people are suffering globally from nearly 80 diverse autoimmune diseases (Selgrade et al., 1999; Cusick et al., 2012). A recent study by American Autoimmune Related Diseases Association (AARDA)^1^ has stated that about 50 million Americans suffer from autoimmune diseases, and women are more likely to be affected than men (www.aarda.org). Therefore, it is very important to understand the mechanisms involved in the breakdown of tolerance in autoreactive T cells and pathophysiology of the autoimmune diseases. Our immune system maintains a finely tuned balance that discriminates between the self and non-self antigens. During thymic education, the majority of the autoreactive T cells are deleted, yet a tiny population escapes and are considered to be responsible for provoking autoimmune diseases. Another well-balanced mechanism known as peripheral tolerance ensures the suppression of remaining autoreactive T cells. Despite the fact that this well-established system operates in our body to eliminate or tolerize autoreactive T and B cells, these cells tend to get activated and inflict debilitating autoimmune diseases (Finnegan et al., 1990; Perola et al., 2002; Hida et al., 2007; Shlomchik, 2008; Mohammed et al., 2011; Cusick et al., 2012; Wang and Zheng, 2013; Curran et al., 2014). T cell-specific autoimmune diseases, such as multiple sclerosis (MS), type-2 autoimmune hepatitis, human type 1 diabetes, primary biliary cirrhosis, meningitis, autoimmune arthritis etc., have been reported in the literature (Perola et al., 2002; Hida et al., 2007; Mohammed et al., 2011; Curran et al., 2014). However, the causative agents are yet to be identified.

Many mechanisms are reported in the literature responsible for the breakdown of self-tolerance due to immunological insult like the aberrant expression of self proteins and exposure of T and B cells to cryptic antigens. Further, an encounter with bacteria, fungi, and viruses, can as well incite molecular mimicry (Fielder et al., 1995; Benoist and Mathis, 2001; McClain et al., 2005). Microbial proteins present in the pathogens may resemble with the host components, thus forming cross-reactive T and B cell epitopes and this may culminate into devastating autoimmune diseases (Wucherpfennig and Strominger, 1995; Birnbaum and Kotilinek, 1997; Birnbaum et al., 1998; Bach, 2005).

The possible role of microbes and their antigenic factors are implicated in several autoimmune diseases (Fujinami and Oldstone, 1985; Krieg, 1995; Bogdanos et al., 2001; Fujinami et al., 2006; Bogdanos and Vergani, 2009; Ercolini and Miller, 2009; Ortega-Hernandez et al., 2010; Cusick et al., 2012; Santiago et al., 2012; Alvarez-Navarro et al., 2013; Doxey and McConkey, 2013; Arleevskaya et al., 2016). In fact, microbial antigens like pulD from *Klebsiella* sp., nuclear antigen-1 from Epstein-Barr virus and OSP-A from *Borrelia* sp. may have a possible association with autoimmune diseases like ankylosing spondylitis, SLE and Lyme arthritis, respectively (Fielder et al., 1995; Benoist and Mathis, 2001; McClain et al., 2005). Furthermore, human and microbial PDC-E2 protein has well-known cross-reactive epitopes that may induce primary biliary cirrhosis (Ortega-Hernandez et al., 2010). Similarly, peptides from DnaJ of microbial protein may bind human HLA-DRB1^*^04:01 molecules and may be involved in the development of rheumatoid arthritis (RA) (Albani et al., 1995). Recently, our group has indicated a probable association of *Mycobacterium tuberculosis* with autoimmune diseases through sequence level molecular mimicry (Chodisetti et al., 2012). Further, T cells reacting with immunodominant epitopes of myelin basic protein (MBP) cross-react with certain viral antigens and therefore may have a likelihood to induce multiple sclerosis (Fujinami and Oldstone, 1985). These reports suggest a possible role of microbial antigens that may provoke autoimmune diseases. Consequently, it may be quite interesting to identify the microbial and human peptides that share cross-reactivity with each other; bind to MHC class II molecules and activate autoreactive T cells. Furthermore, besides sequence, we have taken into consideration structure of the peptides, since it may play a crucial role in imparting autoimmune diseases. Currently, immunoinformatics tools have gained considerable impetus following their implication in various aspects of immunology. For instance, these tools usher a holistic approach to understand the complexity of binding of peptides to various MHC molecules, predicting cytokine release, and identification of T and B cell epitopes (Korber et al., 2006; Tomar and De, 2010).

Based on the above-mentioned facts, we have designed a study to monitor cross-reactivity between microbial and human epitopes and thereby predicted a possibility of the development of autoimmunity. It was interesting to observe that an array of microbial peptides exhibited sequence and structural similarity with human peptides, indicated possible binding to various MHC class II molecules and predicted the production of Th1 and Th2 cytokines. Thus, it can be concluded from the study that there exists cross-reactivity between microbial and human T cell epitopes. Further, this phenomenon may be important in understanding the initiation and prevention of autoimmune diseases.

## Materials and methods

### Programs and databases employed

#### ExPASy

Expert Protein Analysis System (ExPASy) is the well-established proteomics server that allows browsing through several databases (Artimo et al., 2012). The ExPASy server is accessible for many analytical tools to identify protein sequences and their predicted tertiary structures.

#### UniProt

**Figure 1:**
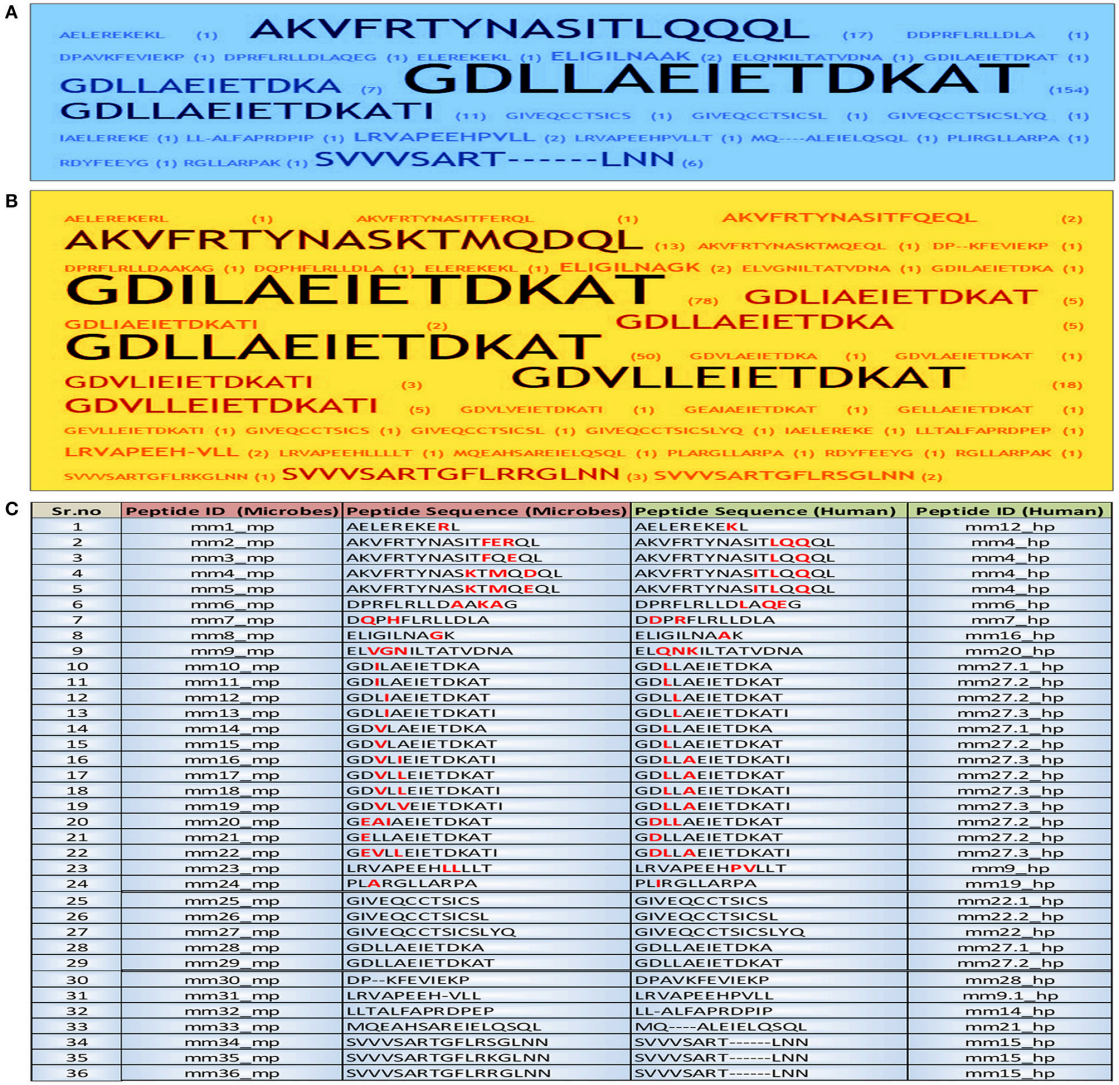
Identification and short listing of peptides exhibiting a degree of molecular mimicry between microbes and human. Word frequency was used for visualizing the occurrence of each peptide in both human and microbes from the initial dataset employing Word Crowd. These sequences were further shortlisted and provided with unique peptide ID. Furthermore, the peptides that appeared more frequently, displayed larger size. Data represents the shortlisted peptide sequences of **(A)** human; **(B)** microbes; **(C)** the total peptide sequences along with ID. The amino acids in each peptide highlighted with red color are the sites of mismatch between the microbial peptide and its human counterpart. mm, molecular mimicry; mp, microbial peptides; hp, human peptides.

UniProt is the central depository of protein sequences and complete catalog of information on proteins, which functions by combining the information contained in UniProt/TrEMBL/Swiss-Prot (Gowthaman et al., 2015). Firstly, we selected the references where published literature describes about autoimmune diseases but antigens are not known (http://www.iedb.org/result_v3.php?cookie_id=6e9073). Next, we identified and analyzed the homologous microbial peptide sequences that are similar to human protein sequences obtained from UNIPROT database. A total of 216 well-characterized peptides of different microbial origin were selected from the database. Further, these sequences were grouped together on the basis of their frequency of occurrence and were provided with unique peptide ID (Figure 1).

The different proteins and their sequences chosen from the literature were compared to their similarity with the human proteome using BLAST program (Altschul et al., 1990) from ExPASy server. Based on the BLAST results, we found regions of 10 or more than 10 sequences of peptides that were similar between the human and microbial proteins, which were selected for further analysis.

The selected human peptides were assessed for binding to HLA DR alleles that are highly prevalent in human population by using the NetMHCII 2.2 Server (Nielsen et al., 2007; Nielsen and Lund, 2009). IC_50_ values were selected based on the binding scores of peptide core regions (10 amino acids length) to each allele. The peptides were classified based on predicted IC_50_ values as strong binders (IC_50_ ≤ 50), weak binders (50 ≤ IC_50_ ≤ 500) and non-binders (IC_50_ ≥ 500) (Nielsen et al., 2007; Nielsen and Lund, 2009). The results were then analyzed by considering the binding affinity of peptides to MHC class II molecules. Also, the nature of antigens, allelic associations, and involvement of different microbes to autoimmune diseases was assessed.

### Establishing binders vs. non-binders

#### NetMHCII 2.2

NetMHCII 2.2 server was used to predict the binding of our peptides to human HLA class II alleles: HLA-DR, HLA-DQ, and HLA-DP. The backend of this algorithm identifies binding affinity of the peptides based on an improved version of neural network alignment procedure and further ranks the binding affinity as strong and weak binders in its output (Nielsen et al., 2007; Nielsen and Lund, 2009). The stand alone software package and the web server are freely available through the URL: http://www.cbs.dtu.dk/services/NetMHCII/.

### Alleles used in the study

Predominantly occurring human HLA class II alleles against which *in silico* prediction can be made are as follows: six DP alleles (HLA-DPA1^*^01:03-DPB1^*^04:01, HLA-DPA1^*^01:03DP B1^*^02:01, HLA-DPA1^*^02:01-DPB1^*^01:01, HLA-DPA1^*^02:01- DPB1^*^05:01, HLA-DPA1^*^01:03-HLA-DPB1^*^03:01_DPB1^*^04: 01, HLA-DPA1^*^03:01-DPB1^*^04:02); Six DQ alleles (HLA-DQ A1^*^01:01-DQB1^*^05:01, HLA-DQA1^*^01:02-DQB1^*^06:02, HLA-DQA1^*^03:01-QB1^*^03:02, HLA-DQA1^*^04:01-DQB1^*^04:02, HLA-DQA1^*^05:01-DQB1^*^02:01, HLA-DQA1^*^05:01-DQB1^*^03:01), and 14 DR alleles (DRB1^*^01:01, DRB1^*^03:01, DRB1^*^04:01, DRB1^*^04:04, DRB1^*^04:05, DRB1^*^07:01, DRB1^*^08:02, DRB1^*^09:01, DRB1^*^11:01, DRB1^*^13:02, DRB1^*^15:01, DRB3^*^01:01, DRB4^*^01:01, DRB5^*^01:01) were selected for this study (Nielsen et al., 2007; Andreatta et al., 2011).

#### IEDB analysis tool

The software suite of IEDB includes the prediction of peptides that bind to MHC class II molecules. The consensus algorithmic approach for prediction of the HLA class II binders includes a combination of NN-align, SMM-align, and combinatorial library methods. IEDB analysis tool predicts peptides binding affinities for 26 allelic variants. The web server can be freely assessed by the URL: http://tools.iedb.org/mhcii/ (Wang et al., 2008, 2010).

#### EpiDOCK

It is a molecular docking based tool that helps MHC class II binding prediction. The tool enables prediction of binding affinity of peptides for 23 most frequent human HLA class II proteins. The structure based algorithmic approach of this tool is based on generating overlapping fragments of nonamer against which modeling and subsequent docking of peptides to each HLA allele are predicted for its promiscuous binding. EpiDOCK can be freely assessed by http://epidock.ddg-pharmfac.net (Atanasova et al., 2011; Patronov et al., 2011, 2012).

### Cytokine secretion profile

#### Il4Pred

IL-4 producing potential of the peptides was checked *via* IL4Pred web server freely available through the URL: http://crdd.osdd.net/raghava/il4pred/index.php. The algorithm discriminates IL-4 inducers and non-inducers by use of machine learning techniques and the binary pattern of the sequences as an input (Dhanda et al., 2013a).

#### IFNepitope

The prediction module of IFNepitope was used to understand the potency of peptides to generate production of the IFN-γ and screen out the positive results. The algorithm of IFNepitope uses the combined strength of motif based techniques and SVM-based learning for discriminating IFN-γ inducing peptides from the negative ones and is freely available through the URL: http://osddlinux.osdd.net/raghava/ifnepitope/index.php (Dhanda et al., 2013b).

### Structure prediction

A peptide structure is highly dynamic in nature and is greatly influenced by the environmental factors present in the vicinity of the peptide. Thus, the best approach for predicting 3D structure of such molecules is *ab initio* based modeling. LOMETS, a local threading meta server was used to generate and explore the tertiary conformational states of each peptide (Wu and Zhang, 2007). LOMETS server is freely available at http://zhang.bioinformatics.ku.edu/LOMETS. The output of LOMETS includes best 10 threading models selected from 160 models by confidence score and as a peptide structure is highly dynamic in nature, all these 10 models have been used in the present study to enhance the confidence interval of the predicted structure.

### Establishing the structural mimicry

Root-Mean-Square-Deviation (RMSD) is commonly used as a metric for measuring the structural similarity of two protein models. In the present study, TM-score was used for establishing the quantitative assessment of the peptides structural similarity based on the RMSD values (Xu and Zhang, 2010).

## Results

The present study was conducted to explore the possibility of microbial peptides that share homology with human proteins in provoking autoimmune diseases by binding to human MHC class II molecules. Consequently, we took a holistic approach to searching and selecting the peptides from the literature using IEDB analysis resources employing desirable fields available at http://www.iedb.org/result_v3.php?cookie_id=6e9073, which are known to be associated with autoimmune diseases (Imai et al., 1995; Rees et al., 1995; Neisser et al., 2000; Nair et al., 2001; Perola et al., 2002; Muratori et al., 2003; Gerster and Dudler, 2004; Peterson et al., 2004; Veeraraghavan et al., 2004; Israeli et al., 2005; Shimoda et al., 2006; Hida et al., 2007; Kawano et al., 2007; Wang et al., 2007; Longhi et al., 2011; Mohammed et al., 2011; Liu et al., 2012; Sonal et al., 2012; Rinaldi et al., 2013; Curran et al., 2014; Rutebemberwa et al., 2014). However, their antigenic sources remain unknown.

### Human proteins that share sequence homology with microbial peptides may predispose toward autoimmunity

We identified from IEDB database sequences that have considerable similarities between the microbial and human peptides based on their *E*-value. The BLAST analysis revealed considerable similarities in sequence level. A total of 216 similar regions of 10 or more amino acids were identified. Further, these sequences were shortlisted according to their frequency of occurrence and were provided with unique peptide ID by peptide frequency employing TagCrowd tool (Figures 1A,B). Noteworthy, the shortlisted sequences displayed molecular mimicry or at least “sequence level mimicry” between human and its microbial peptides. Although, the analysis revealed 24 microbial peptides (Sr. No. 1–24, Figure 1C), largely matched with human peptides but with the certain degree of mismatches. Thus, these peptides were retained for subsequent analysis. Five microbial peptides (Sr. No. 25–29, Figure 1C) showed 100 percent sequence level identity with their human counterparts and 7 peptides (Sr. No. 30–36, Figure 1C) revealed the partial level of homology.

### Microbial epitopes exhibiting cross-reactivity with human peptides show high-affinity binding to MHC class II molecules

**Figure 2:**
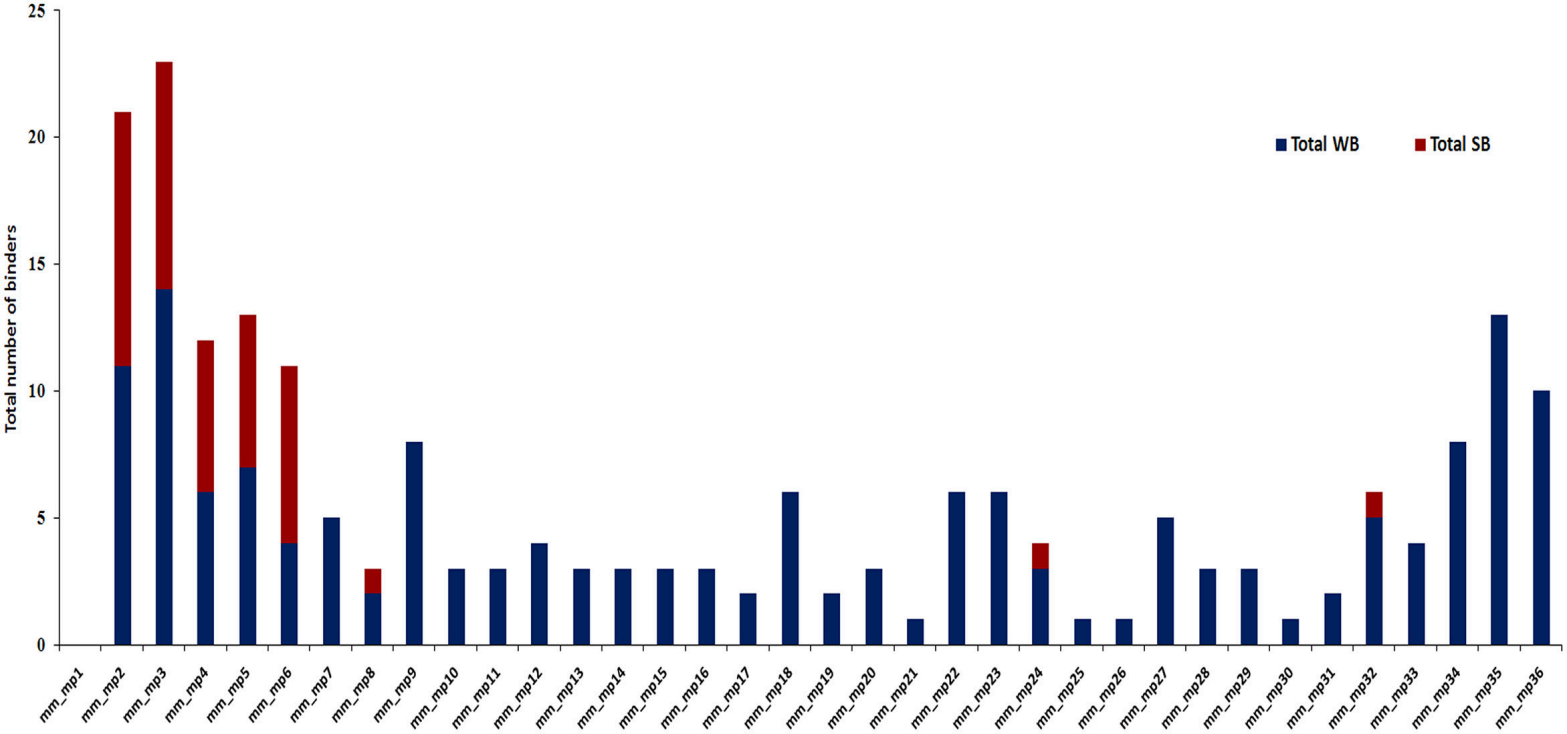
A considerable fraction of microbial peptides displaying sequence homology with human proteins demonstrates strong MHC class II-binding. Bar diagram depicts the number of strong binders (IC_50_ ≤ 50) and weak binders (50 < IC_50_ ≤ 500) utilizing NetMHCII2.2 among the total binders from microbial antigens that shared similarity with human proteins from the identified data set. SB, strong binders (red); WB, weak binders (blue); mm, molecular mimicry; mp, microbial peptides.

**Figure 3:**
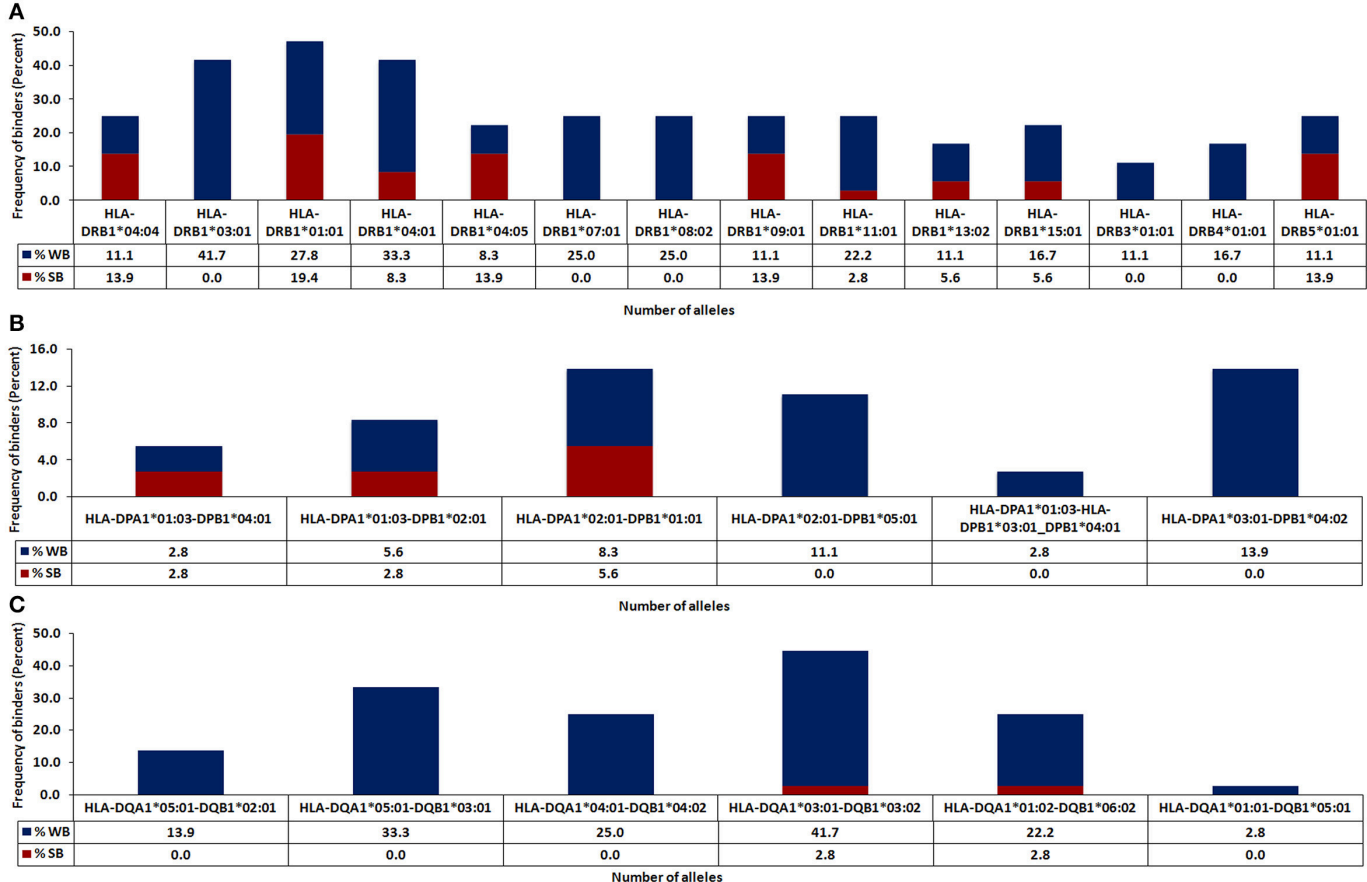
Identification of HLA class II-restricted epitopes of microbial antigens expressing similarity with human proteins. Protein sequences of microbes were subjected to BLAST search with the human proteome for identifying similar regions. The peptides were analyzed for their binding affinity toward the predominantly occurring HLA class II molecules using NetMHCII, as described in material and methods. The peptides were classified based on predicted IC_50_ value as strong binders (IC_50_ ≤ 50), weak binders (50 < IC_50_ ≤ 500) and non-binders (IC_50_ > 500). The total number of binders (strong and weak) for each allele is represented in bar diagram. Data represent the percentage of strong and weak binders for **(A)** HLA-DR; **(B)** HLA-DP; **(C)** HLA-DQ.

The *in silico* analysis revealed microbial peptides that share homology with their human counterparts display differential ability to bind to MHC class II molecules. Peptides with binding scores of IC_50_ ≤ 500 nM were termed as HLA-binders. The peptides with IC_50_ ≤ 50 nM were considered as strong binders (Figure 2). We also found that numerous peptides showed binding to highly prevalent MHC class II molecules viz. HLA-DRB1^*^01:01 (47.2%) followed by DRB1^*^03:01 (41.7%) and DRB1^*^04:01 (41.6%) worldwide (Figure 3A). Notably, people with greater frequency of DRB1^*^01:01, DRB1^*^04:01 and DRB1^*^04:04 are at higher relative risk of microbial pathogenesis and autoimmune diseases like rheumatoid arthritis (Carcassi et al., 1999; Kapitany et al., 2005), haemolytic transfusion reactions (Reviron et al., 2005), psoriasis vulgaris (Cardoso et al., 2005), recurrent respiratory papillomatosis (Bonagura et al., 2004), and rheumatoid vasculitis (Gorman et al., 2004). We could not observe noticeable binding with the other tested alleles (Figures 3A–C). Subsequently, we did an analysis of the strong binders and weak binders among the total epitopes. Even though a greater part of the homologous peptides was found to be as weak binders, a fraction of about 20% was strong binders (Figures 2, 3).

### Microbial peptides demonstrating molecular mimicry with human peptides show promiscuous binding to MHC molecules

**Figure 4:**
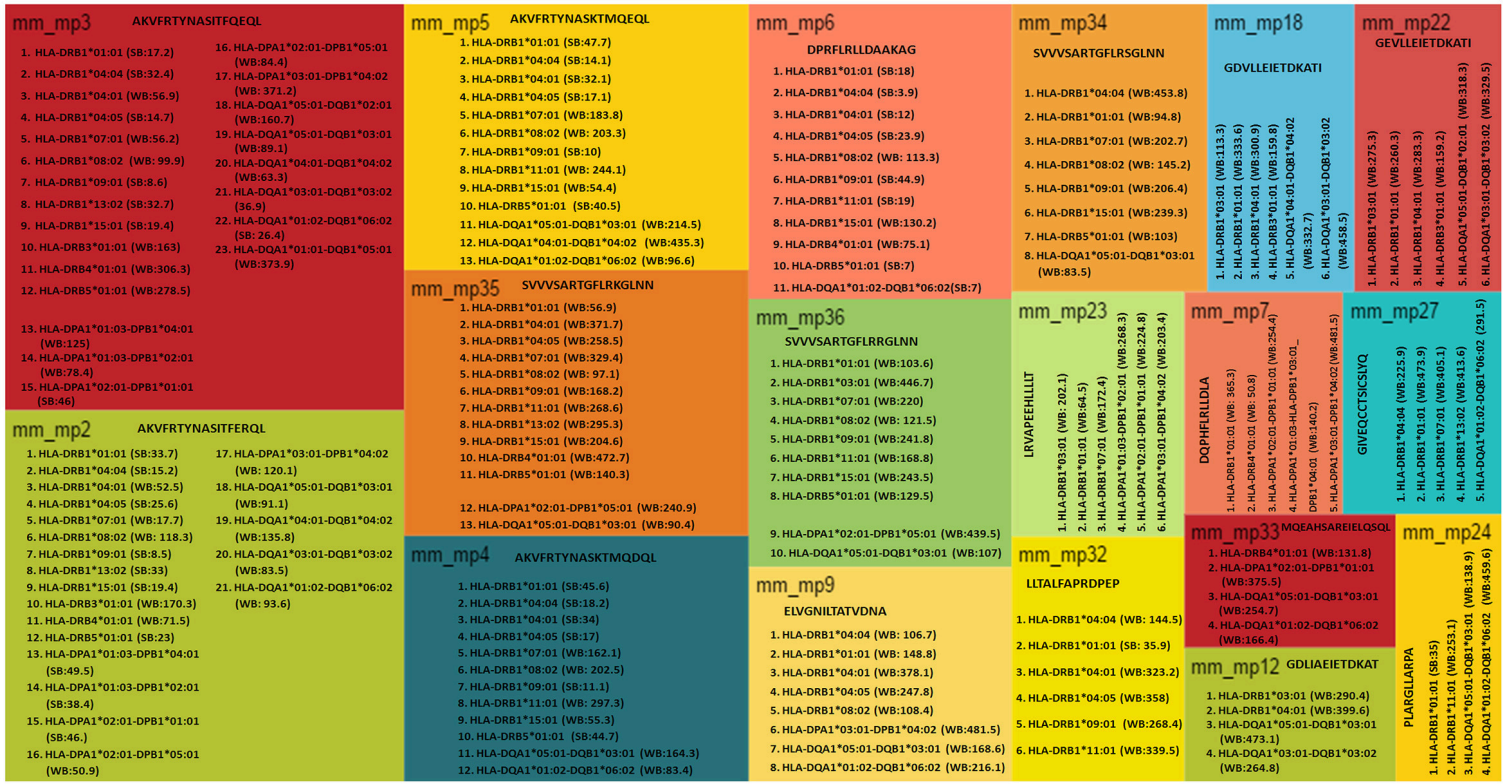
Microbial promiscuous peptides showing sequence homology with human proteins demonstrate strong MHC class II binding. A TreeMap depicting hierarchy among the peptides exhibiting permissive binding for different HLA class II alleles. Each nested rectangle represents different alleles along with its binding score for an array of peptides. Peptides binding to more than three alleles are considered promiscuous in nature.

**Figure 5:**
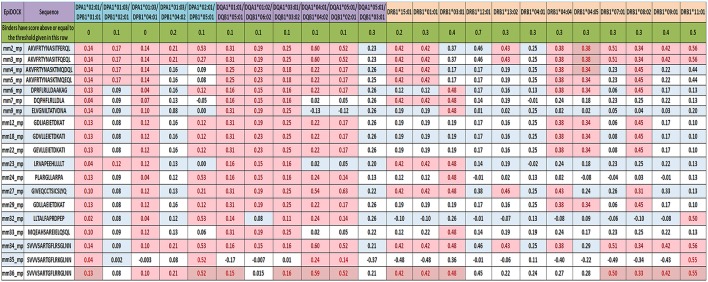
Reaffirming the promiscuity of MHC class II-binding microbial peptides using structural based predicting tool. EpiDOCK, a homology modeling and molecular docking based algorithmic approach for predicting HLA class II binders were used to further check the promiscuity of all the 19 mimicking peptides. A total of 23 most predominant HLA-DP, HLA-DQ, and HLA-DR alleles were used to predict promiscuity. The input sequences were converted into overlapping fragments of 9-mer before deducing the binding score. The fragment of the peptide that showed maximum affinity was considered to be the representative score of mimicking peptide and was indicated in each row. Binders have scored above or equal to the threshold score [(≥0), (≥0.1), (≥0.2), (≥0.3), (≥0.4), (≥0.5), and (≥0.7)] indicated below each HLA allele and highlighted in red color.

**Figure 6:**
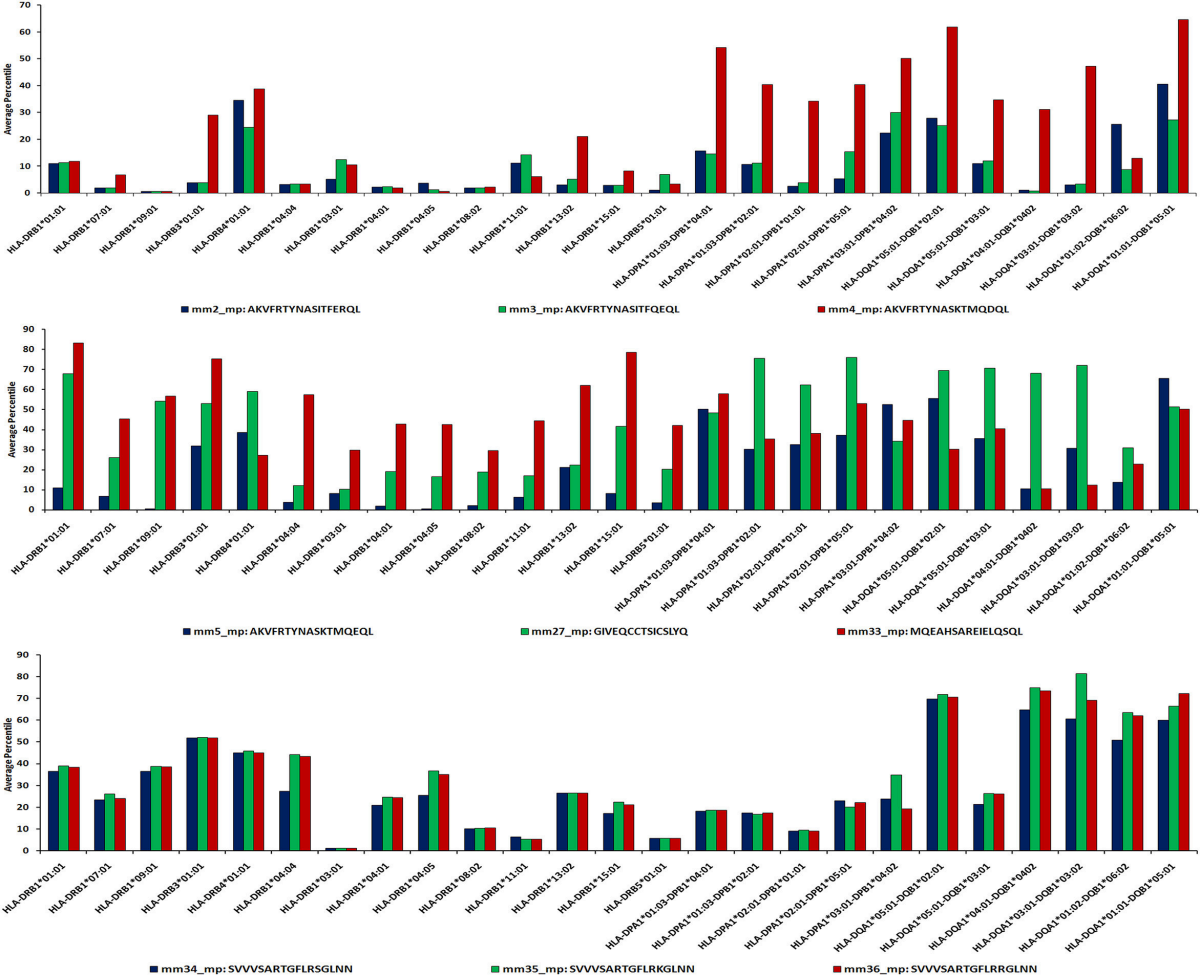
Sequence-based approach reveals promiscuity of MHC class II-binding microbial peptides. IEDB analysis server allows the prediction of HLA binders using sequence-based algorithmic tool for peptides ≥15 mer. The microbial peptide was fragmented into overlapping 15-mer residues and each fragment was predicted for their binding affinity across MHC class II molecules. The score of each fragment was classified based on predicted IC_50_ value as strong binders (IC_50_ ≤ 50), weak binders (50 < IC_50_ ≤ 500) and non-binders (IC_50_ > 500). Based on their IC_50_ values, the peptides are ranked into percentile score for each overlapping fragment. Average percentile score for each fragment represents the overall affinity of the full-length peptide. Low percentile score (≤25) were considered to be HLA class II binders.

Next, we were curious to know whether the microbial peptides that showed sequence similarity with human epitopes were promiscuous binders to various HLA molecules. The peptides that exhibited binding to more than three HLA alleles were selected as promiscuous. Interestingly, AKVFRTYNASITFERQL, AKVFRTYNASITFQEQL, AKVFRTYNASKTMQDQL, and DPRFLRLLDAAKAG sequences were both highly promiscuous and strong binders. Further, among 35 binders, 18 peptides displayed promiscuous binding (Figures 2, 4). To reduce the possibility of false positive and/or negative results, multiple *in silico* platforms based on different algorithmic approaches like sequence (NetMHCII, IEDB) and molecular docking (EpiDOCK) approaches have been applied for the selected peptides. Concurring results across these different predicting algorithms confirmed not only the promiscuity but also the high MHC class II binding affinity of microbial peptides (Figures 4–6).

### Employing NetMHCII, IEDB, and EpiDOCK increases the confidence level of permissive binding of microbial peptides to MHC class II molecules

**Figure 7:**
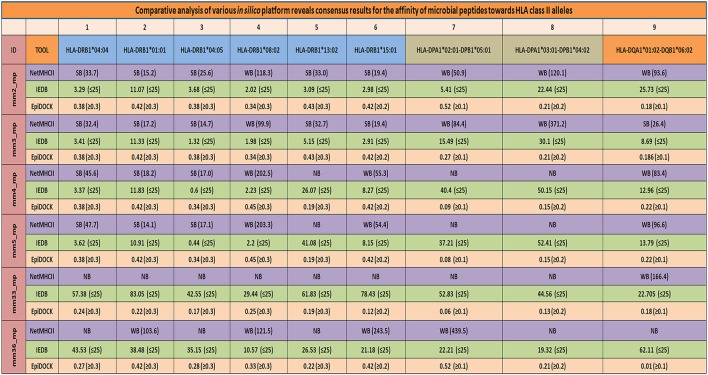
Consensus result using multiple servers confirms the promiscuity of the mimicking microbial peptides. Various *in-silico* platforms (NetMHCII, EpiDOCK, and IEDB analysis resource), using different algorithmic approaches were used to confer the promiscuity of the major mimicking peptides across largely present HLA-DR, HLA-DP, and HLA-DQ alleles. A consensus results in terms of promiscuity of the mimicking peptides were obtained using various *in-silico* platforms which further increased the confidence level of the microbial peptides binding affinity for prevalent HLA class II alleles. SB, strong binders; WB, weak binders; NB, non-binders; mm, molecular mimicry; mp, microbial peptides. NetMHCII: cut-off score SB (IC_50_ ≤ 50), WB (50 < IC_50_ ≤ 500), and NB (IC_50_ > 500); IEDB: binding percentile score (≤25); EpiDOCK: binders have scored above or equal to the threshold value [(≥0.1), (≥0.2), and (≥0.3)] indicated for each of the respective alleles.

To reaffirm the results obtained by NetMHCII, the peptides were subjected to an additional prediction tool EpiDOCK that utilizes a combinatorial method of homology modeling and molecular docking. Additionally, microbial peptides ≥15-mer were checked for their promiscuity employing the IEDB analysis resource. Both EpiDOCK and IEDB generate overlapping fragments of 9-mer and 15-mer residues, respectively. A consensus result was obtained for permissive binding of microbial peptides to various MHC class II molecules by different prediction methods. Thus, endorsing the binding affinity of the peptides across the multiple MHC class II molecules (Figure 7).

### The microbial and human epitopes displaying sequence homology also reveal structural mimicry

**Figure 8:**
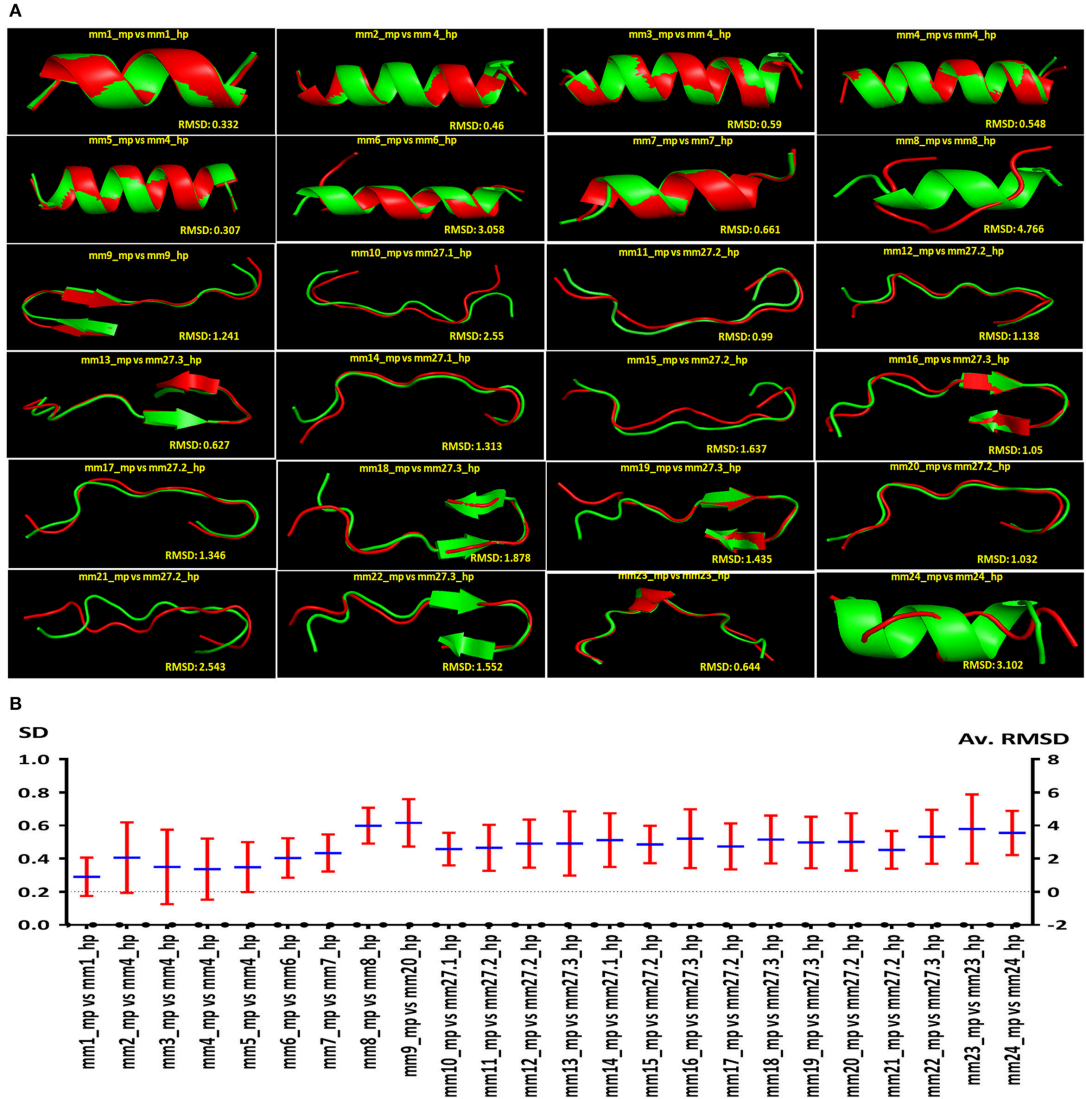
Autoreactive MHC class II binding microbial epitopes showcasing structural similarity with human peptides. Diagram indicating the comparison of predicted structural similarity among the peptides selected from microbes and human. The comparison was made in terms of RMSD. **(A)** The representative (rank one from LOMETS) of the predicted structural similarity between microbial and human peptides. High degree of structural similarity is reflected by the low RMSD value among the structures of autoreactive peptides. **(B)** The average RMSD values and the standard deviation (SD) among the top 10 rank LOMETS predicted structures are represented as point diagram.

The sequence and structure of peptides binding to HLA molecules play a crucial role in the activation of T cells. Consequently, we thought that it would be intriguing to examine whether the peptides showing sequence similarity had a structural resemblance. We obtained 10 comparative structures of putative MHC class II-binding epitopes, ranked according to their RMSD value (Supplementary Figure 1). Further, we shortlisted and presented all the putative MHC class II-binding epitopes and their respective structural level mimicry according to rank 1 (Figure 8A). It was quite interesting to note that the 24 peptides that exhibited likeness at sequence level also portrayed structural mimicry by LOMETS, as evidenced by low RMSD value between microbial and human peptides (Figure 8A). However, there was a varied degree of structural similarity between peptides that had the same sequence. Following observations were made based on the sequence and structural similarity: (i) high sequence and structural identity (mm1_mp, mm3_mp, mm7_mp, mm11_mp, mm13_mp, mm23_mp); (ii) low sequence identity but high structural similarity (mm2_mp, mm4_mp, mm5_mp); (iii) high sequence identity but low structural similarity (mm8_mp, mm10_mp, mm12_mp, mm14_mp, mm21_mp, mm24_mp) (Figures 2, 8B).

### The microbial peptides exhibiting molecular mimicry with their human counterpart show association with autoimmune diseases

**Figure 9:**
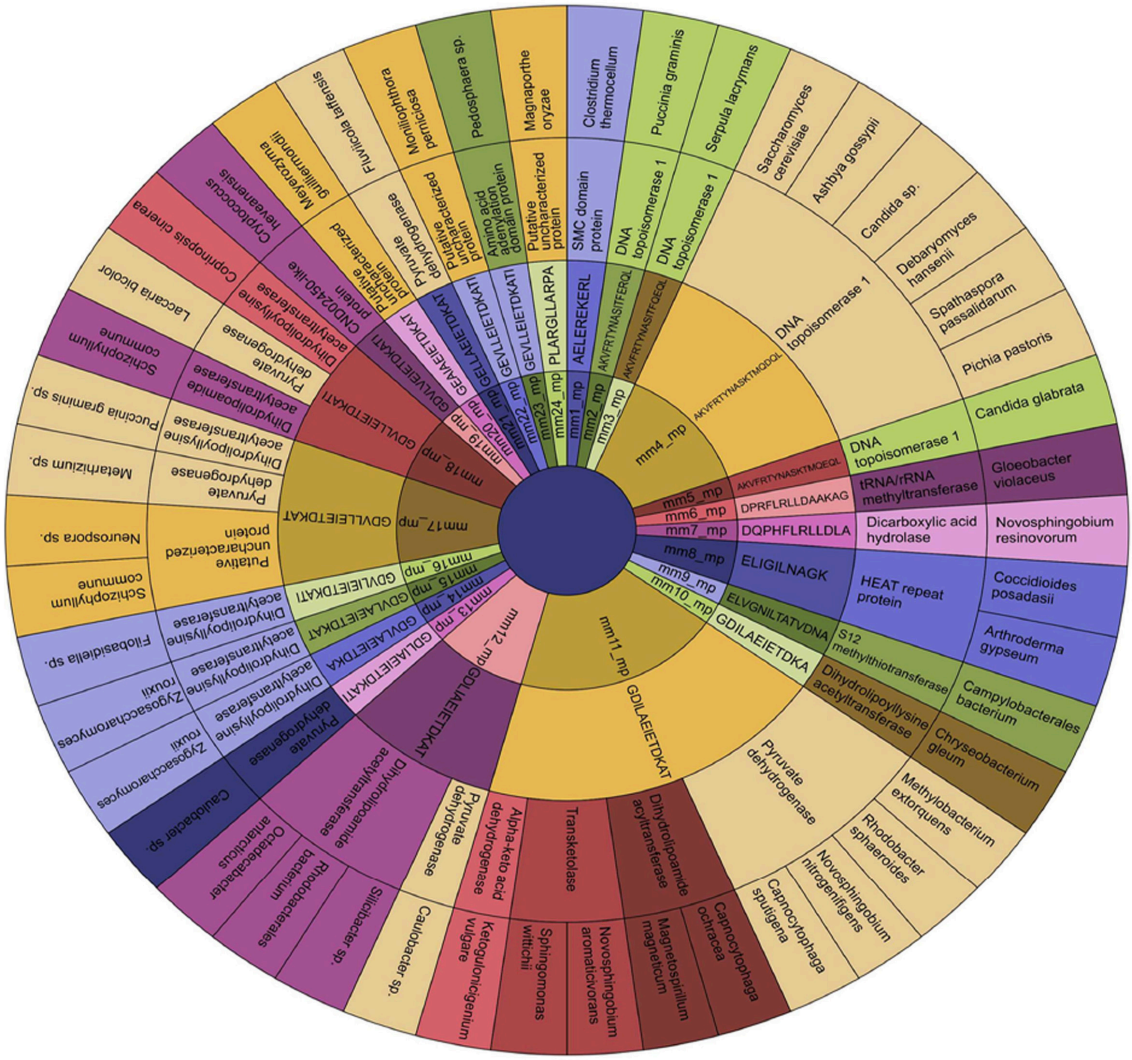
Establishment of the source of the microbial epitopes that show similarity with human peptides. Sunburst Chart representing the microbial peptides and its source protein, and the microbes expressing that protein in a hierarchical pattern. The arrangements of rings are as follows: innermost and subsequent signifies the peptide ID and its sequence, respectively; penultimate ring indicates the protein source of the peptide; final exhibits the microbial species expressing the peptide in that particular protein. The parental node in this figure is the peptide ID and its sequence; whereas the child node is the protein source and the microbes expressing that protein. It is to be noted that few parent nodes (mm4_mp, mm11_mp, mm12_mp, mm17_mp, and mm18_mp) having multiple child nodes are found in different or same proteins present in varied classes of microbes. mm, molecular mimicry; mp, microbial peptides.

The peptides, their protein source and the microbes expressing them were hierarchically clustered into a sunburst chart (Figure 9). The idea behind this was to reveal the relationship among microbial proteins and/or human proteins or the microbes themselves with autoimmune diseases.

**Figure 10:**
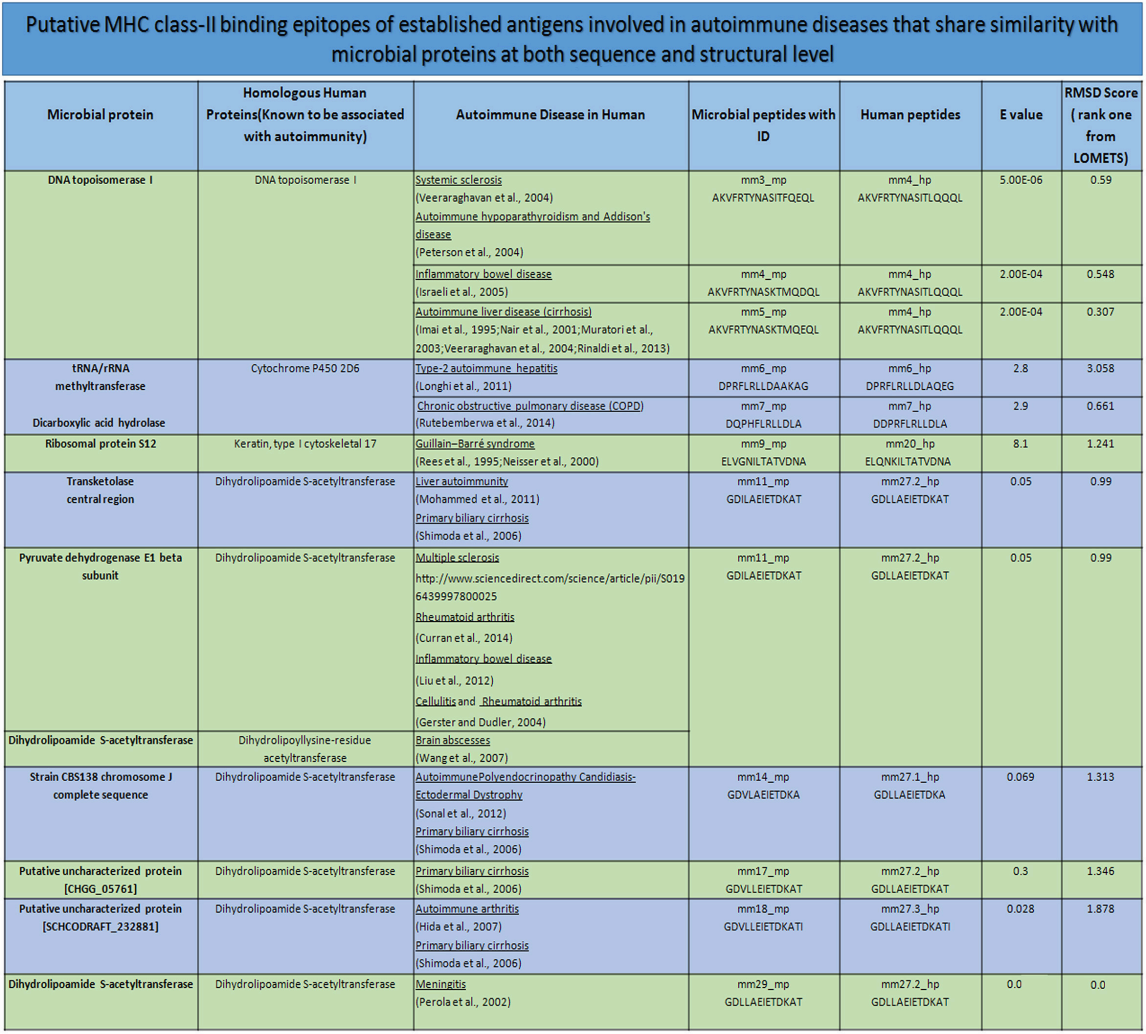
Mimicking peptides and the putative association toward autoimmune diseases. The degree of sequence and structural mimicry observed in human and microbial peptide in terms of *E*-value and RMSD. The record also shows the source of the peptides in human and microbe, along with the autoimmunity associated due to the microbe or the human protein. mm, molecular mimicry; mp, microbial peptides; hp, human peptides; *E*-value, expected value; RMSD, root-mean-square deviation.

Interestingly, we observed few parent nodes (mm4_mp, mm11_mp, mm12_mp, mm17_mp, mm18_mp) that had multiple child nodes, as found in different or same proteins expressed by varied class of microbes (Figure 9). We next addressed whether the host antigens comprising of cross-reactive MHC class II-binding epitopes had any association with autoimmune diseases. Primary biliary cirrhosis, human type 1 diabetes, rheumatoid arthritis and multiple sclerosis are some of the widely known autoimmune disorders affecting a large human population (Tattersall and Gill, 1991; Prince et al., 2004; Kelly and Hamilton, 2007; Scalfari et al., 2013). Many autoimmune diseases are genetically linked but the precise etiology for the onset of these diseases remain unclear (Mariani, 2004; Gregersen and Olsson, 2009). One of the earlier observations suggested the presence of microbial DNA topoisomerase specific T cells in systemic sclerosis, scleroderma and cirrhosis patients (Guldner et al., 1986; Imai et al., 1995; Veeraraghavan et al., 2004). Similarly, we found several peptides exhibiting homology between microbial and human DNA topoisomerase 1, which showed binding to diverse HLA class II alleles (Figures 4, 9, 10). For example peptides AKVFRTYNASITFQEQL, AKVFRTYNASITFERQL, AKVFRTYNASKTMQDQL and AKVFRTYNASKTMQEQL showed promiscuous binding to several HLA class II alleles, including HLA-DRB1^*^01:01, HLA-DRB1^*^04:01, HLA-DRB1^*^04:04, HLA-DPA1^*^02:01-DPB1^*^01:01 and HLA-DQA1^*^03:01-DQB1^*^03:02 with high affinity. Therefore, this observation suggests that there exists a distinct possibility that such cross-reactive epitopes may initiate and instigate the pathogenesis of primary biliary cirrhosis, autoimmune hypoparathyroidism and Addison's disease, systemic sclerosis, inflammatory bowel disease etc. (Nair et al., 2001; Peterson et al., 2004; Veeraraghavan et al., 2004; Shimoda et al., 2006, 2008). Human type 1 diabetes is an autoimmune disease, which has been frequently associated with various microbial and insulin precursor proteins (Lammi et al., 2005; Vaarala et al., 2008). Interestingly, dihydrolipoamide S-acetyltransferase and pyruvate dehydrogenase complex of various microbes are associated in the pathogenesis of primary biliary cirrhosis, chronic active hepatitis (Salaspuro et al., 1976), sarcoidosis (Gur et al., 2007), systemic sclerosis (Whyte et al., 1994), ulcerative colitis (Ohge et al., 2000), and autoimmune hepatitis (Seibold et al., 1996). Our observation also corresponds with this hypothesis (Figures 9, 10).

We identified many MHC class II promiscuous binders from an array of microbial antigens like transketolase, epidermal growth factors, CDA peptide synthetase I, tRNA/rRNA methyltransferase, DNA topoisomerase I, dihydrolipoyllysine-residue acetyltransferase etc. These antigens are associated with insulin-dependent diabetes mellitus, primary biliary cirrhosis, sarcoidosis, type-2 autoimmune hepatitis etc. Some of these antigens exhibited high affinity binding with MHC class II molecules. These findings may signify that the microbial antigens expressing putative autoreactive T cell epitopes exhibit both sequence and structural similarity that may be involved in the progression of autoimmune diseases.

### Cytokine secretion profile of the peptides

**Figure 11:**
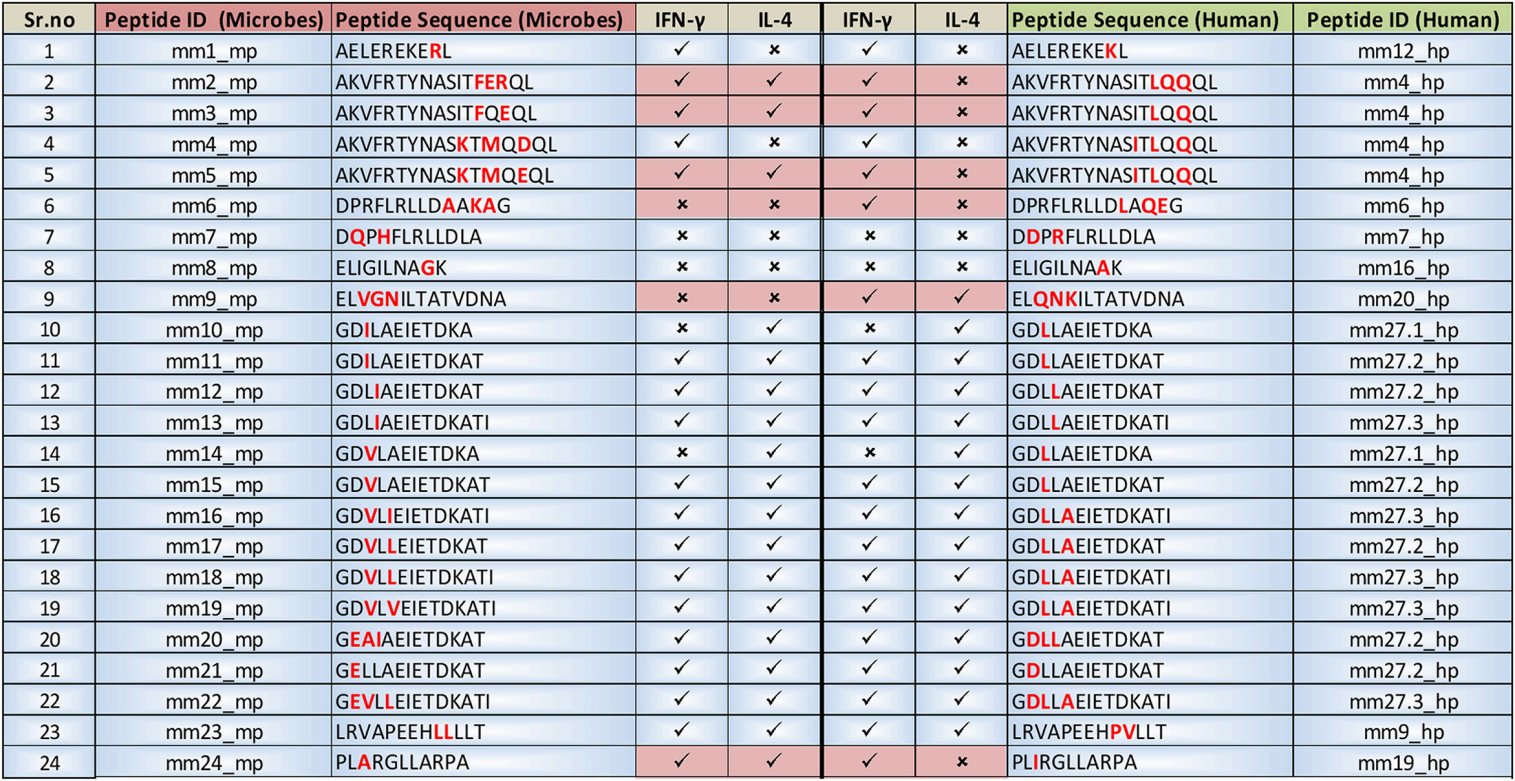
Comparative analysis of the prediction of Th1 and Th2-like cytokines that are elicited by the microbial peptides and human counterparts. IFNepitope and IL4pred servers were used to predict the secretion of IFN-γ and IL-4 for signifying the presence of Th1 and Th2 cells, respectively by the selected microbial and human peptides. The amino acids highlighted in red within the peptide sequence are the sites of mismatch. Data also highlights the peptides illustrating differential IFN-γ and IL-4 cytokine releasing capacity.

Cytokines like IL-12 and IL-4 differentiate naïve CD4 T cells into Th1 and Th2 cells, respectively. In addition, costimulatory molecules, PRRs, peptides interaction with MHC molecules can influence the release of cytokines and the generation of Th1 and Th2 cells (Charlton and Lafferty, 1995; Agrewala and Wilkinson, 1998; Crane and Forrester, 2005). The alteration in the pattern of cytokine secretion induced by the microbial peptides responsible for molecular mimicry may regulate the differentiation of Th1 and Th2 cells. We employed IFNepitope and IL4pred web servers to predict the cytokine secreting ability of the peptides. We noticed that 6 mimicking peptides (mm2_mp, mm3_mp, mm5_mp, mm6_mp, mm9_mp, mm24_mp) differentially induced IFN-γ and IL-4 expression (Figure 11). Elicitation of Th1 and Th2 immunity through molecular mimicry by such peptides may initiate and exacerbate autoimmunity.

## Discussion

Molecular mimicry is based on the fact that T cell and B cell antigenic determinants present in the pathogen may have its counterpart in the host that may possibly trigger autoimmunity (Wucherpfennig, 2001). The concept was first put forward by Fujinami and Oldstone, where they contended that molecular mimicry incited by microbes may contribute to the pathogenesis of MS (Fujinami and Oldstone, 1985). Recently, immunoinformatics tools have helped in various aspects of immunological research, such as in selecting the potential antigenic epitopes, designing *in-silico* vaccine candidates, immune system modeling and developing immunogenic databases, engineering immune therapeutics and designing diagnostic kits. Therefore, in this study, we have analyzed an array of microbial and human peptides that share sequence and structural similarity and studied their implication in molecular mimicry of autoimmune diseases. Following major findings have emerged in context with the present study: (i) an occurrence of a large number of microbial epitopes, which exhibit sequence homology with human proteins that are possibly involved in the triggering of autoimmune responses; (ii) a considerable number of such peptides show promiscuous binding to several MHC class II molecules, which have high relative risks of autoimmune diseases; (iii) microbial components exhibiting cross-reactivity with human peptides show high-affinity binding to MHC class II molecules; (iv) not only sequence alone but the structure of peptides was found to be equally important in ascertaining the molecular mimicry; (v) cross reactive epitopes could differentially activate Th1 and Th2 cells.

Several mechanisms are known to be responsible for autoimmune diseases. One of them is an ancestral pathogenic infection, which leads to a mistaken identity due to the molecular mimicry (Cusick et al., 2012). One of the classical accords for the above hypothesis is the infection with *Streptococcus pyogenes* and as a consequence development of autoimmune acute rheumatic fever (Fae et al., 2006). The study revealed that molecular semblance between the bacterial M protein and human glycoprotein results in a breakdown of self-tolerance and activation of autoreactive T and B cells. These immune cells start attacking the bacterial M protein (Fae et al., 2006), leading to autoimmune diseases known as rheumatic heart disease (Finnegan et al., 1990; Shlomchik, 2008).

Pathogenic organisms have PAMPs (pathogen associated molecular patterns) that are perceived by the immune system as danger signals through pattern recognition receptors (PRRs) (Mogensen, 2009). The pathogen delivers these danger signals along with cross-reactive determinants and activates autoreactive T and B cells. Self-proteins presented by the antigen presenting cells (APCs) cannot activate autoreactive T cells since they lack danger signal. It has been reported that the PRRs licensed APCs display microbial peptides on their surface in context with HLA molecules; resulting in high avidity interaction between autoreactive T cells that eventually break tolerance against self-antigens. Consequently, provoking autoimmune diseases (Mogensen, 2009).

In the past, *in silico* tools have been successfully used to study molecular mimicry in various diseases like tuberculosis, rheumatoid arthritis, multiple sclerosis and ulcerative colitis (Kovvali and Das, 2005; Chodisetti et al., 2012). The present study has identified 36 potential microbial CD4 T cell epitopes that might trigger autoimmunity due to their shared sequence and structural homology with human peptides. Eighteen of such peptides not only exhibited promiscuous binding, but some of them showed high affinity for HLA class II alleles. DRB1^*^01:01, DRB1^*^04:01 and DRB1^*^04:04 HLA alleles are known to be associated with rheumatoid arthritis, haemolytic transfusion reactions, psoriasis vulgaris, recurrent respiratory papillomatosis and rheumatoid vasculitis autoimmune diseases (Carcassi et al., 1999; Bonagura et al., 2004; Gorman et al., 2004; Cardoso et al., 2005; Kapitany et al., 2005; Reviron et al., 2005). Previous studies indicate that polypeptides like Cop 1, glatiramer acetate, Copaxone can suppress the autoreactive T cells and subsequently inhibit the development of autoimmune diseases (Sela, 2006). It may be hypothesized that the T cell epitopes identified in this study can be exploited in designing synthetic peptides that may work in an antagonistic or “batesian mimicry” fashion; thereby inducing immune tolerance in the autoreactive T cells.

In past, mimicry at the level of the sequence of the microbial peptides has been shown to be associated with autoimmune diseases. However, at a cellular level, this mistaken identity actually occurs due to the structural similarity in the MHC class II binding microbial and human peptides. Taking this into account, we have integrated the concept of the involvement of not only sequence but also the structure of the peptide in contributing in the molecular mimicry. Our current findings suggest that both sequence and structure of the peptides are important in contributing in the molecular mimicry.

The cells of the immune system viz. Th17 cells, Th1 cells, Th2 cells, and B cells mainly contribute to the pathogenesis of the autoimmune diseases (Crane and Forrester, 2005; Dorner et al., 2009; Damsker et al., 2010). Currently, the *in-silico* tools are available to identify MHC class II-binding peptides and possible release of cytokines IFN-γ and IL-4 (Dhanda et al., 2013a,b). Th1 cells chiefly secrete IFN-γ and Th2 cells produce IL-4. The role of Th1 and Th2 cells is largely established in many autoimmune diseases. Therefore, in the current study, we have chosen to study the ability of molecular mimicry inducing peptides in releasing IFN-γ and IL-4. As observed in the case of human peptides, their microbial counterparts could elicit the release of same cytokines. Few peptides differentially evoked the secretion of cytokines, as observed from our *in-silico* prediction. The distinct production of cytokines by T cells may be due to the engagement of bacterial PAMPs with PRRs expressed on host APCs during antigen presentation to T cells. It has been reported earlier that the activation and differentiation of Th1 and Th2 cells can occur through the recognition of PAMPs of the microbes by PRRs of the host immune cells (Iwasaki and Medzhitov, 2004; Reis e Sousa, 2004; Mogensen, 2009).

Overall, the study implies that immunoinformatics tools are cost effective, less time consuming, convenient and efficient in identifying microbial peptides that share sequence and structure homology with human peptides. Subsequently, responsible for activating autoreactive T cells and perpetuating autoimmune diseases. Further, identifying the sequence and analyzing the structure of microbial determinants can help in designing potential drug and vaccine targets.

## Author contributions

In this study, the concept designing and the work was done by JA, SP, and DC. The *in silico* procedures were conducted by SP, DC, SN, JK, and BS. The data analysis and manuscript were written by SP, DC, and JA.

### Conflict of interest statement

The authors declare that the research was conducted in the absence of any commercial or financial relationships that could be construed as a potential conflict of interest.

## Footnotes

^1^http://www.aarda.org

## Supplementary material

Supplementary Figure 1 MHC class II-binding epitopes exhibiting structural similarity between human and microbial peptides. Diagram indicating the comparison of ten predicted structural similarity among the autoreactive peptides, selected from microbes (red) and human (green) obtained using LOMETS. Each of the possible structures is compared according to its rank and the RMSD value is indicated.
