# Supplementary figures and images for: Morbid Sequences Suggest Molecular Mimicry between Microbial Peptides and Self-Antigens: A Possibility of Inciting Autoimmunity
Source: Front Microbiol. 2017 Oct 9;8:1938. doi: 10.3389/fmicb.2017.01938 (PMC5640720; doi:10.3389/fmicb.2017.01938)

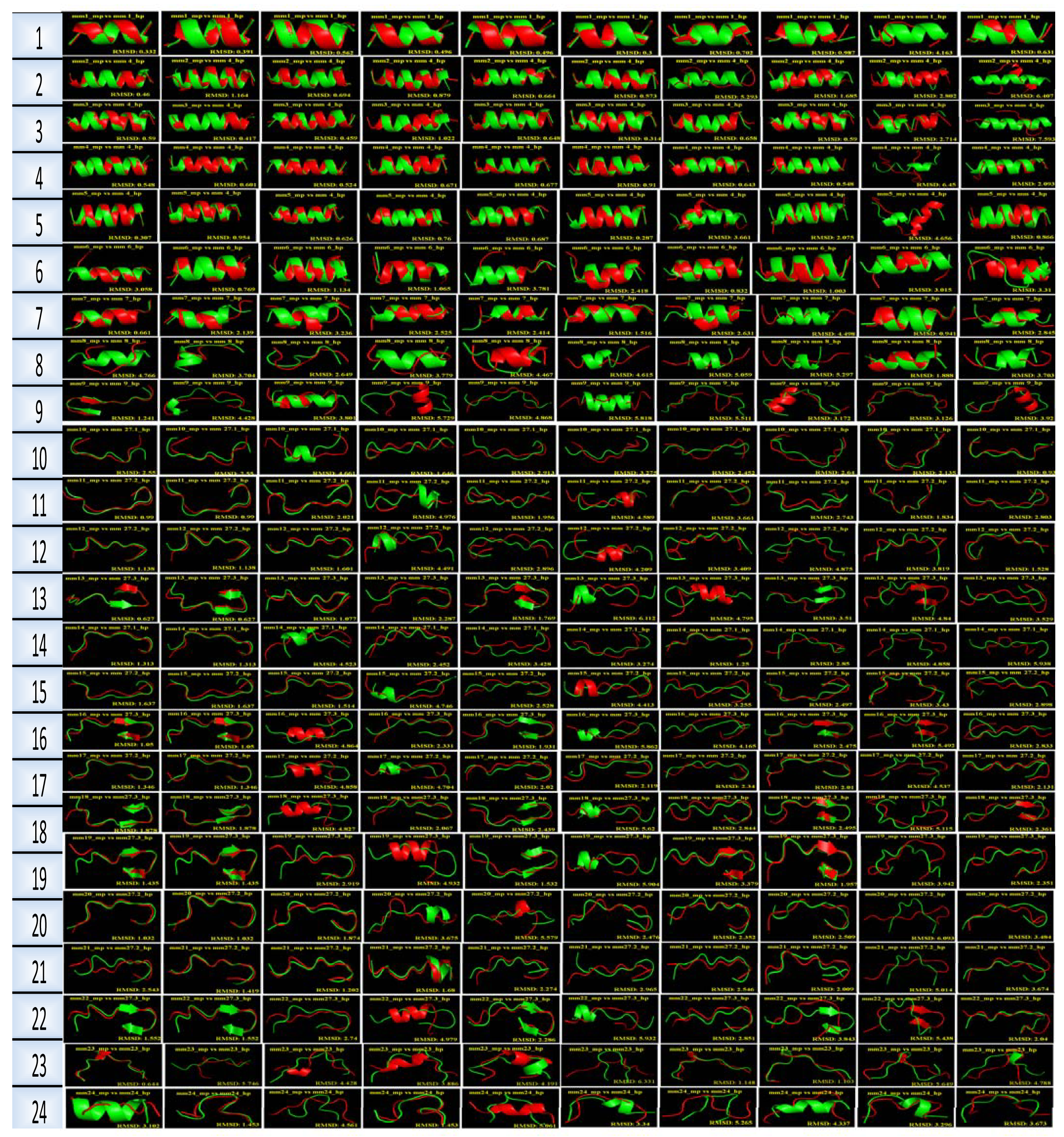

Supplement: Supplementary Figure 1 — MHC class II-binding epitopes exhibiting structural similarity between human and microbial peptides. Diagram indicating the comparison of ten predicted structural similarity among the autoreactive peptides, selected from microbes (red) and human (green) obtained using LOMETS. Each of the possible structures is compared according to its rank and the RMSD value is indicated. [file Image1.TIF]
